# A lightweight, end-to-end explainable, and generalized attention-based graph neural network model trained on high-order spatiotemporal organization of dynamic functional connectivity to classify autistics from typically developing

**DOI:** 10.1162/NETN.a.32

**Published:** 2025-11-20

**Authors:** Km Bhavna, Niniva Ghosh, Romi Banerjee, Dipanjan Roy

**Affiliations:** Department of Computer Science and Engineering, Indian Institute of Technology, Jodhpur, Rajasthan, India; School of Artificial Intelligence and Data Science, Centre for Brain Science and Application, Indian Institute of Technology, Jodhpur, Rajasthan, India; Cognitive Brain Dynamics Lab, National Brain Research Centre, Manesar, India

**Keywords:** Meta-connectivity, Dynamic functional connectivity, Autism, Typical development, Graph-attention neural network, Rough fuzzy C-means algorithm

## Abstract

Autism spectrum disorder (ASD) is a neurodevelopmental disorder characterized by deficits in social cognition, interaction, communication, restricted behaviors, and sensory abnormalities. The heterogeneity in ASD’s clinical presentation complicates its diagnosis and treatment. Recent technological advancements in graph neural networks (GNNs) have been extensively used to diagnose brain disorders such as ASD, but existing machine learning models often suffer from low accuracy and explainability. In this study, we proposed a novel, explainable, and generalized node-edge connectivity-based graph attention neural network (Ex-NEGAT) model, leveraging edge-centric high-order spatiotemporal organization of dynamic functional connectivity streams between large-scale functional brain networks implicated in autism. Using the Autism Brain Imaging Data Exchange I and II datasets (total samples = 1,500), the model achieved 88% accuracy and an F1-score of 0.89. Additionally, we used meta-connectivity subtypes to identify subgroups within ASD samples using the rough fuzzy c-means algorithm. We also used connectome-based prediction modeling, which revealed critical brain networks contributing to predictions that accurately correlate with Autism Diagnostic Observation Schedule (ADOS) and full intelligent quotient (FIQ) scores. The proposed framework offers a robust approach based on previously unexplored higher order spatiotemporal correlation features of dynamic functional connectivity, which may provide critical insight into ASD heterogeneity and improve diagnostic precision.

## Author Summary

In this study, we proposed a novel, explainable, and generalized node-edge connectivity-based graph attention neural network model, leveraging edge-centric high-order spatiotemporal organization of dynamic functional connectivity streams between large-scale functional brain networks implicated in autism. The connectome-based prediction modeling reveals critical brain networks contributing to predictions that accurately correlate with ADOS and FIQ scores. The proposed framework offers a robust approach based on previously unexplored higher order spatiotemporal correlation features of dynamic functional connectivity, which may provide critical insight into autism spectrum disorder heterogeneity and improve diagnostic precision.

## INTRODUCTION

Autism spectrum disorder (ASD) is a lifelong pervasive neurodevelopmental disorder that impairs a variety of functions, among which an individual’s social cognition and communication deficits are the most prominent symptoms (American Psychiatric Association, 2013; Shao, Fu, You, & Fu, 2021). In particular, individuals with ASD face several challenges in their day-to-day lives and often acquire concurrent disorders such as depression, anxiety, and attention-deficit/hyperactivity disorder (ADHD), which may further complicate the clinical diagnosis, particularly in younger children (Almuqhim & Saeed, 2021). While no accurate treatment for autism is currently available, early detection may be advantageous in alleviating primary symptoms of autism by providing appropriate interventions (Almuqhim & Saeed, 2021; American Psychiatric Association, 2013).

Functional magnetic resonance imaging (fMRI) is an effective method that has provided a deeper understanding of the pathophysiology of ASD (Kennedy & Courchesne, 2008). According to previous studies using fMRI, it has been found that autism primarily affects the abilities of social cognition, interaction, and communication abilities that are associated with specific alternations in the functional brain networks such as theory of mind (ToM), default mode network (DMN), central executive network (CEN), and salience network (SN) (Bandara & Riccardi, 2024; Bhavna, Akhter, Banerjee, & Roy, 2024; Bhavna, Banerjee, & Roy, 2023; Hong et al., 2019; Kennedy & Courchesne, 2008; Müller & Fishman, 2018; Parisot et al., 2018).

In recent years, significant advancements have been made in identifying neuroimaging-derived biomarkers related to ASD through resting-state fMRI (rs-fMRI) analysis. Abnormal brain regions, such as the cingulate gyrus and precentral gyrus, have been linked to ASD, with various studies employing statistical methods and machine learning (ML) to classify and predict ASD based on functional connectivity (FC) and characterizing FC subtypes for stratification of ASD (Bi, Zhao, Xu, Sun, & Wang, 2018; Ren et al., 2023). In addition, the precentral gyrus, occipital lobe, thalamus, anterior cingulate gyrus, superior temporal gyrus, and praecuneus were discovered to play important roles in the classification of ASD (Z. Wang, Xu, Peng, Gao, & Lu, 2023). While traditional approaches often relied on small sample sizes and handcrafted features characterizing group-level differences in FC, dynamic functional connectivity (dFC), and their variability among specific large-scale brain networks (Harlalka, Bapi, Vinod, & Roy, 2019; Hong et al., 2019; Nair et al., 2020), recent developments in deep neural networks (DNNs) now leverage big data and artificial intelligence (AI) frameworks to automatically generate effective features from fMRI data, enhancing early diagnosis prospects (Nogay & Adeli, 2020; Xu et al., 2020). Furthermore, the recent accessibility of large neuroimaging datasets opens up excellent opportunities to develop data-driven DNN models for hypothesis-driven and exploratory studies (Di Martino et al., 2014). Previous works have successfully integrated ML techniques for feature selection (Li et al., 2019; Li, Liu, Tang, & Lei, 2020) and employed algorithms like Support Vector Machine (SVM) and convolutional neural networks (CNNs) for dealing with computationally aided diagnosis of ASD (Eslami, Mirjalili, Fong, Laird, & Saeed, 2019; Liu, Xu, Li, Yu, & Yu, 2020). Beyond ML models, deep learning models, such as CNNs, are an effective and scalable approach for distinguishing between ASD and Typically developing (TD) without requiring manual feature engineering (Supekar et al., 2022). Although CNN operates well with gridlike input in Euclidean space, such as naturalistic images, it is probably unsuitable for use with non-Euclidean data, such as brain imaging (Rosenbaum, Smith, Kohn, Rubin, & Doiron, 2017). Consequently, the geometric distance between distinct brain areas in Euclidean space may not effectively reflect the functional distance. Some geometric deep learning approaches, such as graph neural networks (GNNs), may circumvent this constraint and better cope with non-Euclidean data types like graphs (Xia et al., 2021; Zhou et al., 2020). The previous studies used GNN and attention-based GNN for ASD classification in two ways: either using node-centric features (Bandara & Riccardi, 2024; Parisot et al., 2018), in which FC along with phenotypic features (e.g., age, gender, and ASD scores) were used to train the model at the group level, or using graph-based features set (Gao et al., 2023; Yao et al., 2021), in which node-centered local information (or in other words, each sample was treated as a graph) was used to train the model. This GNN architecture has performed modestly due to the following limitations. In many of the previous approaches, the models mainly focus on static or node-centric features in spatial space, so the importance of edge-centric features (FC links) depicting relationships between meta-links is largely ignored (Chen et al., 2024; Parisot et al., 2018). Secondly, due to the high dimensionality of the GNN features, significant symptomatic variations, and heterogeneity of data across different sites, the previous GNN models gave low reliability, robustness, and generalizability. Lastly, the internal architecture of GNN models takes time to execute information from one node to another, which increases the execution time to process data (Xia et al., 2021; Zhou et al., 2020). These limitations collectively contribute to overfitting the classification model and lead to unsatisfactory identification performance on independent datasets (Chen et al., 2024). Hence, there is a genuine need for an explainable GNN architecture that can utilize both edge- and node-centric features given by standard FC as well as ASD-associated alterations of dFC and spatial covariation features of FC links captured by meta-connectivity (MC), along with the sufficient generalizability of the proposed model architecture.

There is a genuine knowledge gap in the extant literature on how the correlation between FC links (known as MC) between the key large-scale brain networks that constitute higher order spatiotemporal correlation patterns undergoes alteration during atypical neurodevelopment in ASD (Arbabyazd et al., 2020; 2023; Battaglia et al., 2020; Betzel, Faskowitz, & Sporns, 2023). MC quantifies how fluctuations in different FC links covary over time. Conceptually, it captures the higher order temporal coordination between dynamic FC patterns, offering a richer spatiotemporal representation of brain network organization beyond traditional static or even first-order dynamic FC. In this vision, MC is still a graph, although over a broader set of nodes than the original FC graph, and any usual graph-theoretical method valid for standard networks can also be applied to MC. Previous work has further shown that dFC modules tend to include star subgraphs with well-defined center regions—meta-hub—and divergent links (Arbabyazd et al., 2020; Battaglia et al., 2020), which tend to be all strong or all weak simultaneously, depending on time. These meta-hubs act as “puppet masters,” moving the “threads” in specific dFC modules and reaching regions that can be far away and potentially distributed brain-wide. A recent study by Arbabyazd et al. (2020) demonstrated that resting-state fluctuations of different functional links are not independent but are constrained by high-order correlations between triplets or quadruplets of functionally connected regions and are precociously degraded in Alzheimer’s disease. Hence, these higher order correlations between triplets or quadruplets of functionally connected regions underlie important biological functions in the context of development and disease. Additionally, metalinks between large-scale functional brain modules may serve as a set of important spatiotemporal features that can be used for training ML models to classify ASD from TD. This approach may provide more nuanced insights about atypical FC MC patterns of several functional brain networks implicated in social cognition and communication deficits. To the best of our knowledge, little is known about their functional implications and role as potential biomarkers for ASD classification, which is the key issue we tackle in this work by developing a novel framework.

This study presents three main contributions: (a) We hypothesized that metalinks between ToM, DMN, CEN, and SN could serve as imaging biomarkers to differentiate autism from TD individuals. We calculated MC matrices to capture cofluctuations across these networks, providing insights into spatial relationships and temporal dynamics. (b) We proposed a novel, explainable, and generalized node-edge connectivity-based graph attention neural network (Ex-NEGAT) model to track higher order correlations in resting-brain dynamics while reducing computational requirements. By applying a depth-first search (DFS) approach, we trained the model on the Autism Brain Imaging Data Exchange (ABIDE) I dataset and tested it on the ABIDE II dataset, utilizing leave-one-out and fivefold cross-validation to validate results and minimize overfitting. The Ex-NEGAT model improves upon traditional GNNs by emphasizing node and edge interactions within an attention framework. It uses a spatiotemporal MC matrix to capture dynamic connectivity patterns important for time-dependent biomarkers, enhancing ASD classification accuracy and interpretability compared with typical node-centric models.

**Figure 1.**
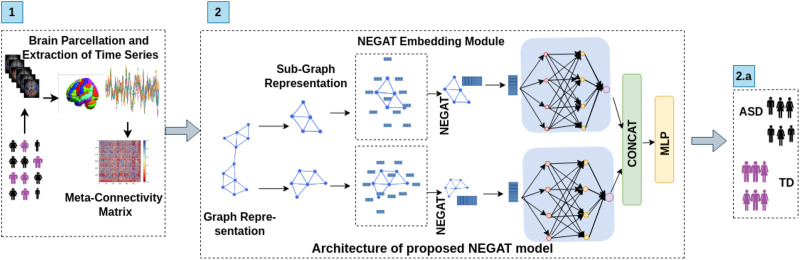
Illustrative overview of lightweight, generalized proposed Ex-NEGAT framework for classifying ASD from TD using meta-connectivity as an edge-centric feature set. Step 1 involves BOLD time-series signal extraction and calculation of meta-connectivity matrices; Step 2 shows the details of the proposed architecture of the NEGAT framework to perform classification.

To identify the uniqueness of the MC feature as a higher order correlation among triplets or quadruplets, we used the rough fuzzy c-means (RFCM) algorithm to stratify ASD subgroups. This approach employed fuzzy membership to assign samples to clusters, categorizing them into lower and upper approximate sets based on membership degrees. We tested the reliability of MC subtypes as predictive biomarkers by predicting symptom severity scores for participants using connectome-based prediction modeling (CPM). Our generalized Ex-NEGAT model outperformed previous GNN models in classifying ASD from TD individuals, accurately identifying subgroups, and predicting symptom severity. This framework reduces execution time for computing MC and training/testing GNN models, addressing challenges in diagnosing ASD by identifying salient functional connectivity links in large-scale brain networks (refer to Figure 1).

## RESULTS

### Computation of MC Matrices

**Figure 2.**
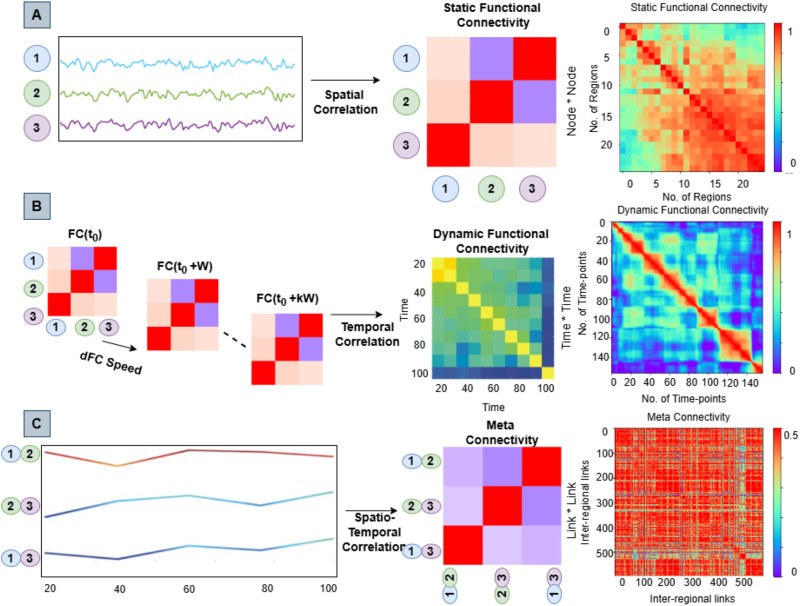
Calculation of static, dFC, and MC. In the traditional approach (A), neural activity is averaged over time to create an FC matrix. We calculated a dynamic FC stream using shorter sliding windows (B), leading to a dFC matrix. In (C), we considered each FC link as a dynamic variable, giving rise to an MC matrix by capturing covariances between these dynamic links.

In the current study, we extracted time-series signals from ToM, DMN, CEN, and SN brain networks and calculated functional connectivity matrices for each subject in both groups. The FC matrices have a unique symmetry, and despite their large *N* × *N* size, they only have *L* = *N*(*N* − 1)/2 unique entries, representing the lower triangular part of the matrix. As the output, the FC matrix appears as an *N* * *N* matrix. We also calculated the dFC stream that outputs in a 3D tensor-sized *N* * *N* * *F*, where *F* was the number of frames. We employed a sliding window approach to estimate dFC, using a window length of 20 s with a step size of 2 repetition time (RT), aligned with the ABIDE dataset. This approach offered a balanced trade-off between capturing transient neural dynamics and ensuring reliable correlation estimates within each window. While we also tested a 30-s window, the window size = 20-s configuration gave more robust classification results (Table 1), likely due to its ability to capture faster temporal fluctuations relevant to ASD characteristics, consistent with prior studies (Allen et al., 2014; Demirtaş et al., 2016; Rashid, Damaraju, Pearlson, & Calhoun, 2014). However, each FC matrix was shown in the “vector” format that outputs a vector of size *L* * 1, and the dFC stream yielded a 2D matrix sized *L* * *F*, with each frame in vector format. The “vector” format is advantageous because it produces more memory-efficient output, which is essential for large datasets. It also naturally represents FCs as points in a vector space, making it easier to create dimensionally reduced representations of the dFC stream using methods like t-stochastic neighborhood embedding. After that, we calculated MC matrices for each individual of size 600 * 600, as displayed in Figure 2. We performed multiple one-sample *t* tests with a *p* value < 0.01 and applied false discovery rate correction to validate the results. We performed statistical tests to validate the MC matrix, which serves as a preprocessing step and is not central to the GNN’s core functionality. While statistical filtering helps remove noisy or irrelevant connections, GNN architecture plays a crucial role in learning the complex relationships between regions of interest (ROIs) and significantly enhances performance. Statistical filtering ensures that the GNN is not overwhelmed by insignificant or spurious connections, but the main contribution comes from how the GNN processes these significant edges to capture more intricate patterns.

To assess the robustness of the MC matrices to noise, Gaussian noise with varying signal-to-noise ratios, ranging from 20 dB to −5 dB, was added to the data. MC matrices were then computed from sliding-window dFC matrices. The similarity between the noise-free and noisy MC matrices was evaluated using Pearson correlation and the Structural Similarity Index Measure (SSIM). A controlled simulation was conducted in which structured ground-truth MC matrices were systematically perturbed with increasing levels of Gaussian noise. Results showed that the MC matrices retained their core structure and clustering features under low to moderate noise levels (*SD* = 0.01–0.2), with Pearson correlation values ≥ 0.86 and SSIM values ≥ 0.69. However, significant degradation of the MC matrices was observed at higher noise levels (*SD* = 0.5).

**Table 1.** Performance of proposed Ex-NEGAT model using FC, dFC, and MC matrices as feature set with *d* = 3, where W.s represents window size

**Sr. no.**	**Feature set**	**Samples (train/test)**	**Accuracy**	**F1-score**	**Recall**	**Precision**	**Execution time (s)**
1	FC	840/660	63%	0.62	0.58	0.59	120
2	dFC (W.s = 30 s)	610/475	70%	0.71	0.66	0.68	170
3	dFC (W.s = 20 s)	840/660	74%	0.73	0.74	0.71	198
4	MC (W.s = 30 s)	610/475	83%	0.85	0.84	0.81	217
5	MC (W.s = 20 s)	840/660	**88%**	**0.89**	**0.87**	**0.88**	**235**

### Classification of ASD and TD Individuals Using Proposed Ex-NEGAT Model

#### Performance of proposed method using MC matrices.

**Figure 3.**
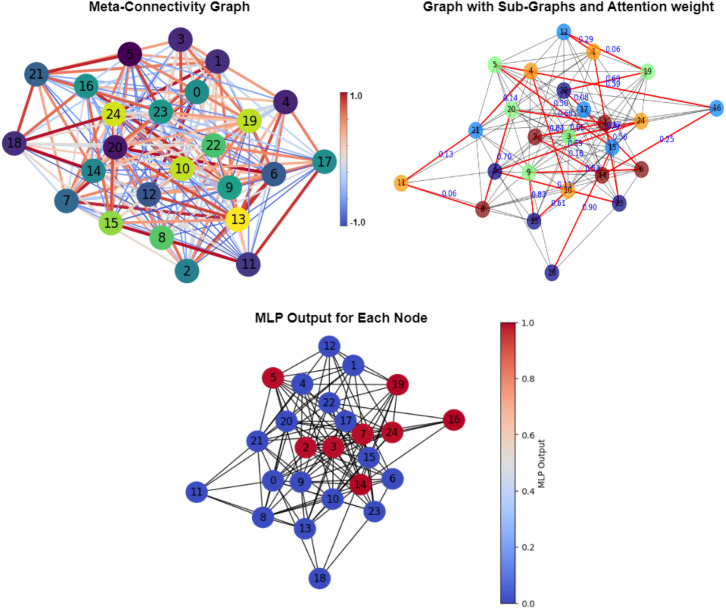
The figure illustrates the working of the proposed model. It shows how the MC matrix has been converted into a graph, then a subgraph with attention weight has been extracted, and the final multilayer perception (MLP) graph is computed.

**Figure 4.**
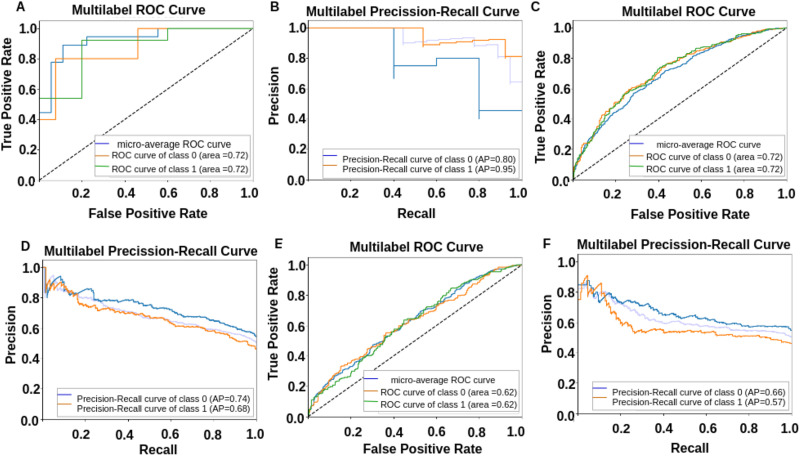
(A) and (B) are depicting classification results using MC with window size = 30 s. (C) and (D) are depicting classification results using MC with window size = 20 s. Whereas (E) and (F) are depicting classification results using FC. (G) and (H) are showing classification results using dFC.

**Figure 5.**
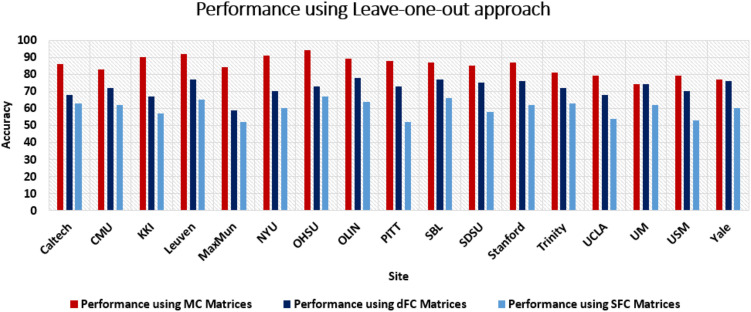
Performance of proposed model using leave-one-out approach. The results show that the proposed model outperforms when trained on the proposed MC as a feature set compared with FC and dFC measures.

In the current study, we proposed an attention-based GNN in which we divided the graph into a subgraph up to a maximum depth *d* using the DFS approach, which helped to identify a suitable value for depth *d* (refer to Figure 3). We empirically evaluated subgraph depths from *d* = 2 to *d* = 5 and found that *d* = 3 offered the best trade-off between performance and efficiency, that is, high accuracy with less execution time. Deeper values beyond *d* = 3 did not improve classification performance and instead increased computational overhead and redundancy (refer to Table 2). So, we reported all the results using the value of *d* = 3. We selected a depth *d* = 3 for the proposed model based on a balance between performance, computational efficiency, and model complexity. While *d* = 4 yielded slightly better results, the performance improvement was marginal compared with the increased computational cost. A higher depth often introduces a greater risk of overfitting, especially with limited datasets, so we opted for *d* = 3 as a more efficient yet effective architecture. To classify ASD samples from neurotypical, we subjected resting-state MC matrices of size 600*600 to the proposed Ex-NEGAT model. Here, we considered and performed the complete analysis using window sizes of 20 and 30 s. Firstly, we performed normal training and testing processes on the ABIDE 1 dataset and achieved an average accuracy of 92% with an F1-score of 0.94 for a window size of 20 sec and an average accuracy of 91% with an F1-score of 0.90 for a window size of 30 sec. To make the model generalizable, we trained the model on the ABIDE 1 dataset and performed testing on the ABIDE II dataset. We achieved 88% average accuracy with an F1-score of 0.89 for a window size of 20 s and 83% average accuracy with an F1-score of 0.85 for a window size of 30 s. The window size of 20 s yielded better results (refer to Figures 4 and 5 and Table 2). We have also conducted an ablation study to isolate the effects of statistical significance testing and other model components and reported the results in Table 6.

**Table 2.** Performance of proposed model using different depth values *d*

**Value of** *d*	**Performance using FC matrices**	**Performance using dFC matrices**	**Performance using MC matrices**
	**Acc. (%), F1-s, Prec., Rec., Exe. (s)**	**Acc. (%), F1-s, Prec., Rec., Exe. (s)**	**Acc. (%), F1-s, Prec., Rec., Exe. (s)**
*d* = 1	54%, 0.53, 0.54, 0.51, 80	65%, 0.67, 0.63, 0.64, 120	71%, 0.73, 0.68, 0.67, 156
*d* = 2	59%, 0.61, 0.57, 0.59, 103	69%, 0.67, 0.68, 0.66, 159	76%, 0.77, 0.71, 0.72, 197
*d* ** = 3**	**63%, 0.62, 0.58, 0.59, 120**	**74%, 0.73, 0.74, 0.71, 198**	**88%, 0.89, 0.87, 0.88, 235**
*d* = 4	66%, 0.67, 0.63, 0.65, 135	73%, 0.76, 0.72, 0.70, 220	89%, 0.87, 0.88, 0.85, 329
*d* = 5	65%, 0.65, 0.63, 0.66, 176	75%, 0.73, 0.76, 0.71, 308	87%, 0.85, 0.83, 0.81, 433

The table highlights accuracy, F1-score, precision, recall, and execution time in seconds.

#### Comparison of performance using MC with state-of-the-art feature sets such as static and dFC.

An extensive comparative analysis assessed the proposed model’s performance, leveraging the novel MC feature set against the conventional ones, for example, resting-state functional connectivity (rs-FC) and dFC patterns. This entailed the execution of classification tasks using both static and dFC. The Ex-NEGAT model was trained using rs-FC matrices derived from ToM, DMN, CEN, and SN on the ABIDE I dataset, with subsequent testing on the ABIDE II dataset. We achieved an average accuracy of 63% with an F1-score of 0.62 using FC matrices on unseen data. We again trained the Ex-NEGAT model using each individual’s dFC on the ABIDE I dataset. We tested the DFC of the ABIDE II dataset. We achieved an average accuracy of 70% with an F1-score of 0.71 for a window size of 30 s and an average accuracy of 74% with an F1-score of 0.73 for a window size of 20 s. We have obtained better results using a window size of 20 s. There was an improvement in performance using dFC matrices as a feature set compared with rs-FC as a feature set (refer to Table 2). These results collectively highlight the limitations of conventional feature sets in effectively classifying the ASD samples when confronted with unseen data. In contrast, the MC matrices exhibited remarkable performance as a feature set that emphasizes higher order correlation, capturing edge strength variations over time for each individual (refer to Table 3). When applied to unseen data, it successfully distinguished the ASD population in the context of specific brain networks. This underscores the efficacy of the proposed framework in leveraging MC matrices as a feature set for specific brain networks, illuminating a promising avenue for further exploration.

**Table 3.** Performance comparison of multiple models, including the proposed model, in classifying ASD from neurotypical samples using MC matrices as a feature set

**Sr. no.**	**Classifier**	**Accuracy**	**F1-score**	**Recall**	**Precision**	**AUC**
1	SVM	50%	0.48	0.46	0.45	0.52
2	Random forest	56%	0.55	0.53	0.54	0.55
3	LSTM-RNN	71%	0.72	0.69	0.70	0.69
4	2d-CNN	75%	0.74	0.73	0.72	0.76
5	CapsNet	78%	0.78	0.76	0.770	0.76
6	Graph convolutional network (GCN)	71%	0.69	0.67	0.70	0.73
7	Chebyshev Convolution (GCNN)	75%	0.74	0.73	0.75	0.76
8	Residual GCN (ResGCN)	76%	0.77	0.75	0.76	0.73
9	Graph SAGE	80%	0.81	0.78	0.77	0.82
10	Proposed Ex-NEGAT	**88%**	**0.89**	**0.87**	**0.88**	**0.86**

#### Cross-validation using fivefold cross-validation and leave-one-out approaches.

The classification framework’s evaluation involves utilizing two distinct approaches to mitigate overfitting and ensure a robust and generalized assessment of its classification performance. The initial approach entails implementing fivefold cross-validation across all subjects, with a specific value of *k* (in this study, *k* = 5). The second approach adopts a more stringent method known as the leave-one-out approach. Under this strategy, one particular site’s data are designated as an independent testing dataset, while the data from all other sites collectively constitute the training dataset. Using fivefold cross-validation, we achieved an average accuracy of 81% with an F1-score of 0.78 for a window size of 30 s and an average accuracy of 82% with an F1-score of 0.83 for a window size of 20 s. While using the leave-one-out method, we achieved an average accuracy of 78% with an F1-score of 0.79 for a window size of 30 s and an average accuracy of 85% with an F1-score of 0.84 for a window size of 20 s. We observed that leave-one-out gave better generalized results in which each site’s data were treated as unseen data for testing (refer to Table 4 and Figure 5). Table 5 displays top 10 pairs of nodes whose edge connectivity contributed most to the prediction accuracy. We have also conducted an ablation study to isolate the effects of statistical significance testing and other model components and reported the results in Table 6.

**Table 4.** Validation of performance of the proposed EX-NAGET model using the leave-one-out approach

**Site**	**No. of samples**	**No. of M/F**	**Performance using MC matrices**	**Performance using dFC matrices**	**Performance using FC matrices**
			**Accuracy (%), F1-score**	**Accuracy (%), F1-score**	**Accuracy (%), F1-score**
Caltech	43	29/14	86%, 0.84	68%, 0.69	63%, 0.62
CMU	37	31/6	83%, 0.85	72%, 0.73	62%, 0.61
KKI	65	46/19	90%, 0.91	67%, 0.66	57%, 0.59
Leuven	85	65/20	92%, 0.90	77%, 0.78	65%, 0.67
MaxMun	72	64/8	84%, 0.85	59%, 0.60	52%, 0.53
NYU	312	240/72	91%, 0.89	70%, 0.72	60%, 0.61
OHSU	39	39/0	94%, 0.92	73%, 0.73	67%, 0.68
OLIN	48	38/10	89%, 0.88	78%, 0.77	64%, 0.65
PITT	76	63/13	88%, 0.90	73%, 0.73	52%, 0.53
SBL	40	40/0	87%, 0.84	77%, 0.73	66%, 0.68
SDSU	45	34/11	85%, 0.87	75%, 0.72	58%, 0.55
Stanford	47	35/12	87%, 0.86	76%, 0.73	62%, 0.64
Trinity	71	71/0	81%, 0.83	72%, 0.74	63%, 0.60
UCLA	138	113/25	79%, 0.82	68%, 0.67	54%, 0.55
UM	211	173/38	74%, 0.77	74%, 0.73	62%, 0.63
USM	94	94/0	79%, 0.78	70%, 0.72	53%, 0.54
Yale	77	55/22	77%, 0.79	76%, 0.76	60%, 0.63

**Table 5.** Table shows the top 10 pairs of nodes whose edge connectivity contributed most to the prediction

**Sr. no.**	**Brain region pairs**	**Connectivity strength (ASD)**	**Connectivity strength (TD)**
1	ACC-MPFC	0.53	0.35
2	Precuneus-LLPFC	0.65	0.43
3	RPPC-PCC	0.42	0.64
4	LTPJ-RTPJ	0.71	0.36
5	vlPFC-LSTS	0.29	0.49
6	Precuneus-LANG	0.27	0.48
7	LLPFC-LPPC	0.41	0.23
8	LLP-LIPC	0.35	0.51
9	RLP-RTPJ	0.27	0.38
10	RSTS-RANG	0.41	0.24

The table shows edge connectivity strength for ASD and TD groups.

**Table 6.** Performance of proposed model using ablation study, which shows the effect of each parameter (hyperparameter, turning off attention mechanism, etc.) on the model’s performance based on the estimation of accuracy, precision, recall, and F1-score

**Model configuration**	**Accuracy**	**Precision**	**Recall**	**F1-score**	**Explanation**
Baseline GNN (no statistical testing)	60%	0.58	0.56	0.58	Standard GNN model, without statistical testing
GNN + statistical testing	66%	0.65	0.63	0.64	Statistical filtering of meta-connectivity
GNN + meta-connectivity (no testing)	71%	0.72	0.69	0.70	Meta-connectivity without statistical filtering
GNN + hyperparameter (tuned)	77%	0.74	0.73	0.75	Tuned hyperparameters, no statistical testing
Full model (hybrid approach)	**88%**	**0.89**	**0.87**	**0.88**	Hybrid model: statistical testing, meta-connectivity, GNN, tuned hyperparameters
Full model (no attention mechanism)	78%	0.79	0.77	0.79	Remove attention mechanism
Full model (no MLP layers)	75%	0.74	0.73	0.75	Remove MLP layers

### Identification of ASD Subgroups Using RFCM

We analyzed a total of 711 ASD samples, including data from ABIDE I and ABIDE II, to uncover hidden subgroups within the ASD population, aiming to better understand the heterogeneity within it. We applied the RFCM in which MC matrices were subjected as input. Our analysis revealed three distinct subgroups: the first subgroup included 361 samples, the second subgroup included 76 samples, and the third subgroup included 274 samples. To identify subgroups within ASD samples using clinical scores, the previous study (Sadiq, Al-Hiyali, Yahya, Tang, & Khan, 2022) reported three main conditions within ASD: autistic disorder (ATD), Asperger’s disorder (APD), and pervasive developmental disorder (PDD) not otherwise specified. ATD is characterized by significant impairments in social interaction, communication, stereotyped behaviors, and interests, typically manifesting before age 3 years and often accompanied by language delays and intellectual disabilities. APD involves challenges in social interaction, nonverbal communication, and repetitive behaviors, but typically without language or cognitive development delays. PDD is diagnosed when an individual shows some but not all symptoms of ATD or APD or has milder symptoms not fully meeting the criteria for either condition. To validate the result, we explored the association between clinically defined ASD subgroups and subgroups identified using MC between brain regions. We found that: (a) the first subgroup, characterized by severe ASD, included 199 individuals with ATD, 66 with APD, and 14 with PDD. (b) The second subgroup, showing moderate ASD severity, included 155 individuals with ATD, 51 with APD, and 20 with PDD. (c) The third subgroup, more closely associated with typical development, included 37 individuals with ATD, 13 with APD, and 156 with PDD.

#### Clustering methodology.

**Figure 6.**
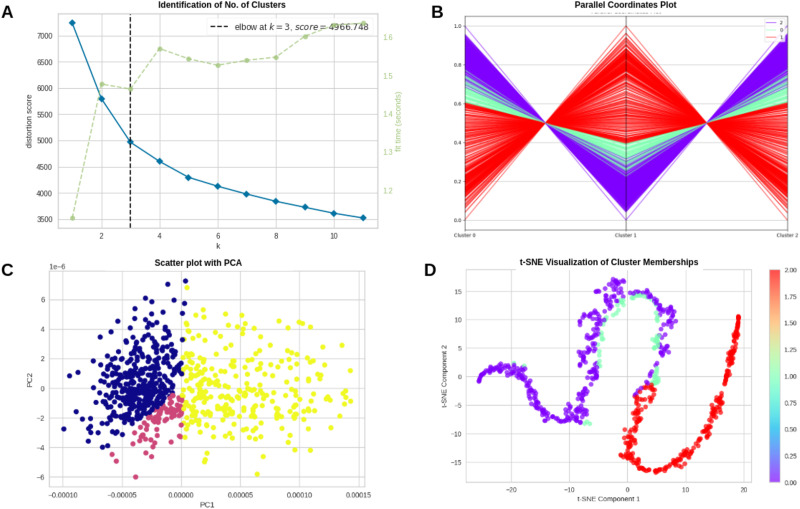
(A) identifies the number of clusters using the elbow approach. (B), (C), and (D) show visualizations of clusters using different approaches.

In this study, we applied RFCM to partition ASD individuals into *n* = 3 distinct clusters based on their MC matrices extracted from the mentioned brain networks. The number of clusters was identified using the elbow approach shown in Figure 6A. The algorithm was configured with a fuzziness coefficient of *m* = 2, an error threshold of 0.005, and a maximum of 1000 iterations to ensure convergence (refer to Figure 6).

#### Cluster characteristics.

The clusters were characterized by their centroids, representing the average connectivity patterns within each cluster. The centroids were computed using fuzzy membership values (*μ*_*ij*_), where higher values indicate stronger membership to a particular cluster. This approach allowed us to capture each sample’s gradual degrees of belongingness to multiple clusters, reflecting the complex nature of ASD subgroups.

#### Lower and upper approximation.

**Figure 7.**
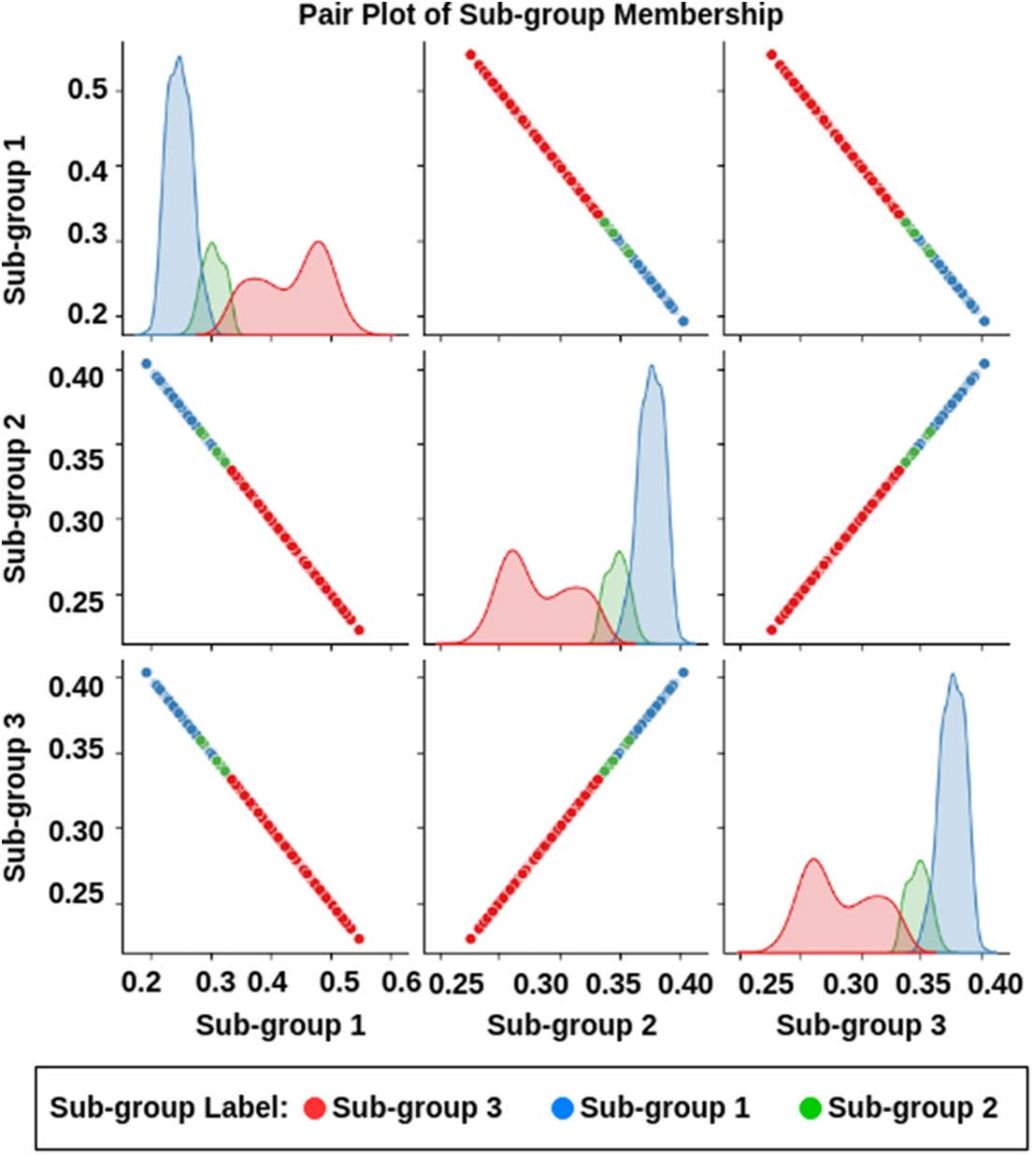
Illustrating the association between all three clusters, that is, how one sample in a cluster is also associated with other clusters with a membership confidence value. The result suggested that Subgroup 2 was highly associated with Subgroups 1 and 3, whereas Subgroups 1 and 3 were slightly associated with each other.

In RFCM, each cluster is defined by its lower approximation (*R*(*X*)) (We define the lower approximation threshold at *μ*_lower_ = 0.3, indicating that samples with membership degrees *μ*_*ij*_ ≥ 0.3 are considered definitively belonging to cluster *X*_*i*_.) and upper approximation (*R*(*X*)) (The upper approximation threshold is set at *μ*_upper_ = 0.6, including samples with membership degrees 0.6 ≤ *μ*_*ij*_ < 0.3 in the cluster *X*_*i*_). These samples exhibit some similarity with the cluster but do not meet the higher certainty threshold of the lower approximation (refer to Figure 7). The lower approximation includes objects that belong to the cluster, while the upper approximation encompasses objects that may or may not belong, considering the uncertainty in membership assignment. This dual approximation framework provides a nuanced understanding of cluster boundaries and membership uncertainties within the ASD population.

#### Validation of results.

To assess the validity of the identified clusters, we calculated several cluster validity indices: (a) silhouette score: 0.65, (b) Davies-Bouldin Index (DBI): 0.42, and (c) Dunn Index: 2.1. These indices measure the cohesion within clusters and separation between clusters. A higher silhouette score, Dunn Index, and a lower DBI suggest well-defined and distinct clusters in our analysis.

#### Interpretation of cluster patterns.

To visually explore the identified clusters, Figure 7 presents a pair plot of cluster memberships. Each scatter plot depicts the relationship between membership values across pairs of clusters, with KDE plots along the diagonal showing the distribution of membership values within each cluster. The colors denote cluster labels, illustrating how individuals are distributed across the identified subgroups based on their connectivity profiles. Interestingly, the first subgroup contained unique samples with lower associations with the other subgroups. In contrast, the second subgroup exhibited a high degree of association with the first subgroup and a moderate association with the third subgroup (refer to Figure 7). Additionally, some samples showed associations with all three subgroups. Lastly, the third subgroup was slightly associated with the first subgroup. The first and third subgroups showed unique samples with less association in the overall analysis.

#### Analysis of each cluster.

**Figure 8.**
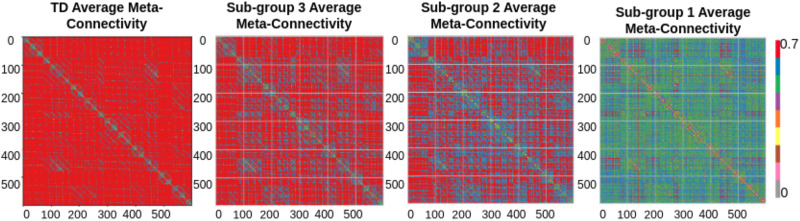
Average MC matrices for neurotypicals, Subgroup 1, Subgroup 2, and Subgroup 3 that show that Subgroup 3 is closer to neurotypicals and less associated with ASD symptoms. Whereas Subgroup 1 is more associated with ASD symptoms.

To gain further insight into our results, we calculated the mean connectivity matrix for each subgroup. Notably, the third subgroup displayed a notably high correlation (0.7, *p* < 0.02) between its edges, while the second subgroup yielded a similar result, with a high correlation (0.6, *p* < 0.03) (refer to Figure 8). Conversely, the first subgroup demonstrated a moderate correlation (0.5, *p* < 0.01) between its edges. For validation purposes, we conducted multiple one-sample *t* tests on each connectivity with a significance level set at *p* < 0.01 and applied FDR correction. We also calculated the mean MC matrix for the TD group for comparison purposes and observed a high correlation between edges in the TD group. We found that the third subgroup was more associated with the TD group, whereas the second subgroup was moderately associated, and the first subgroup was less associated with the TD group.

### Prediction of Symptom Severity Score

**Figure 9.**
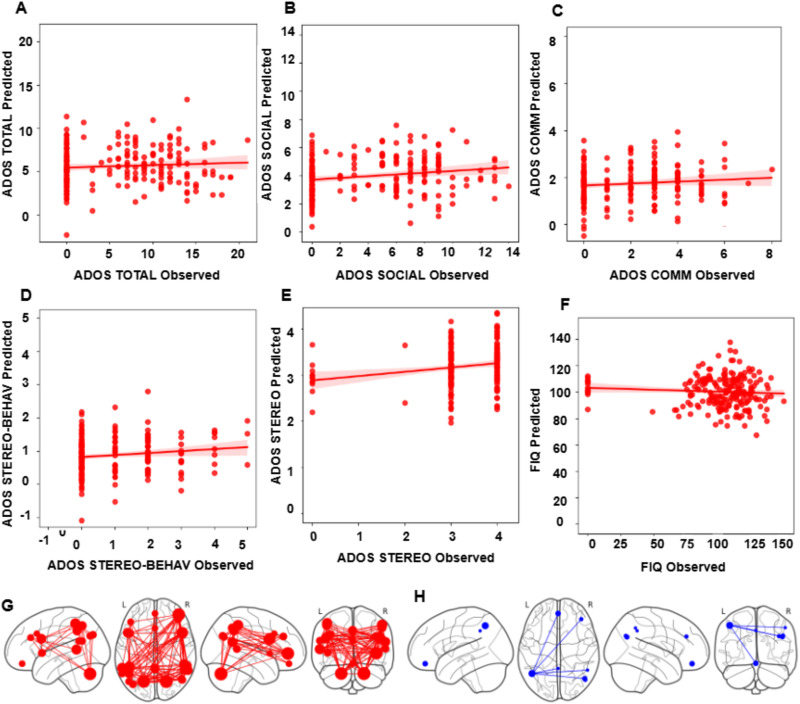
(A–F) Scatter plots of observed versus predicted scores for various ADOS and cognitive measures. The red line represents the linear regression fit between the predicted and observed values, while the shaded area indicates the 95% confidence interval. Pearson correlation coefficients (*r*) between observed and predicted scores are displayed in the lower right corner of each plot. (G) represents the nodes whose connectivity contributed positively to prediction, whereas (H) represents the models whose connectivity contributed negatively to prediction.

To identify the robustness of MC matrices as a higher order correlation feature set, we predicted symptom severity scores for each ASD sample using the CPM approach. We used MC matrices of the ABIDE I dataset for training the model and performed testing on unseen data, that is, MC matrices of the ABIDE II dataset. Our proposed model gave an average accuracy of 85% with an F1-score of 0.86. We also performed validation using a fivefold cross-validation approach. Our model predicted ADOS-Module, ADOS-Stereo, ADOS-Communication, FIQ, and ADOS-Total scores more accurately, whereas it did not give accurate results for ADOS-Social scores. We also predicted symptom severity scores using FC matrices. We got an average accuracy of 75% with an F1-score of 0.76 using FC matrices. Our approach is novel as no framework in the literature has identified higher order correlation (MC matrices) as a predictive measure for the prediction of symptom severity scores (refer to Figure 9). Even in large testing data, the proposed framework could predict symptom severity scores up to a considerable range.

## DISCUSSION

In this work, we developed an Ex-NEGAT model that could capture resting-state fluctuations of different functional links in ASD compared with TD individuals. The fundamental assumption was that these meta-links are not independent but are constrained by high-order correlations between triplets or quadruplets of functionally connected brain regions during atypical neurodevelopment and can be considered a correlate of cognitive processing. Conceptualizing dFC as a flow across morphing connectivity configurations, our notion of dFC speed quantifies the rate at which FC networks evolve in time, which serves as an important imaging biomarker for accurate classification between ASD and TD and predicting symptom severity scores at the individual subject level based on MC subtypes. Here, we probe the hypothesis that variations of resting state dFC flow characterized by MC are selectively interrelated within specific functional subnetworks (ToM, DMN, CEN, and SN networks) and associated with deficits in social cognition, communication, and interaction abilities frequently reported in the extant literature (Shao et al., 2021; Supekar et al., 2022). Focusing on a selective subset of networks well motivated by existing works by avoiding the unnecessary complexity of whole brain parcellation, which results in reduced computational costs and enhances efficiency while capturing key aspects. While correlations between these networks have already been explored, the true novelty of our work is demonstrating how high-order correlations between triplets or quadruplets, nonlinear interactions between functional networks that evolve dynamically over time. Unlike first-order correlations, which only capture pairwise interactions, higher order correlations provide a multi-dimensional view of network interactions, revealing how functional networks cofluctuate at different temporal resolutions and capturing subtle yet critical differences in network coordination in ASD.

### Advantages of Using MC Feature to Discover New ASD Biomarkers

A growing body of recent works, including previous works from our group, indicates that aberrations in ASD are frequently associated with FC and dFC between salient brain regions (Arbabyazd et al., 2020; Harlalka et al., 2019). Many similar studies using feature selection based on graph data have been carried out for other mental disorders such as schizophrenia and ADHD (Demirci et al., 2008). According to the existing literature, large-scale brain networks anchored in ToM, DMN, CEN, and SN networks and their connectivity profiles based on structural and functional neuroimaging to index atypical development in autistic individuals subserve as crucial biomarkers of brain graphs (Marshall et al., 2020; Mouga et al., 2022). However, to our knowledge, the brain’s functional network entails connectivity and dynamics. Large-scale brain network dynamics during connectivity switching driven by various internal states importantly attributes neural flexibility and the mutual influence observed between graph-edges over time or time-dependent functional links (Brovelli et al., 2017; Faskowitz, Esfahlani, Jo, Sporns, & Betzel, 2020). Hence, it is highly probable that MC between these subnetworks may also exhibit mutual influences, a notion rarely addressed in research. Identifying new biomarkers related to diseases holds significant importance for early diagnosis and treatment.

### Advantages of Using MC Matrix and Attention-Based GNN

One frequently employed method to apply traditional ML algorithms or artificial neural networks to graph data is to ignore the graph’s structure and treat the input edge weights as a vector of features (Munsell et al., 2015). However, the caveat with many of these approaches is that they ignore the crucial topological and statistical relationship among functional brain modules and large-scale brain networks, which is important for the emergent properties of brain functions and constraining dynamics (Kawahara et al., 2017). Another recent model has introduced a Spatial–Temporal Attention Graph Convolutional Network to classify functional connectivity (Wang, Kong, Hou, Yang, & Yuan, 2022). In the spatial domain, this model utilizes attention-enhanced graph convolutional networks (GCNs) to process the topological features of brain regions. It employs a multihead self-attention method in the temporal domain to capture the temporal relationships between different dFC (Wang et al., 2022). Although many recent attempts use the temporal and spatial characteristics of fMRI data, the accuracy of ASD diagnosis leaves something more to be desired. It falls well short of the expected classification accuracy (W. Wang et al., 2022; Z. Wang et al., 2023). An alternative approach could be to treat the graph’s adjacency matrix as an image and extract features using traditional CNN models. However, as shown, the spatial proximity in the adjacency matrix elements does not always correspond with the topological locality of the graph data (Z. Wang et al., 2023). However, in this work, we differed from all the previous approaches and used a node-edge-centric spatiotemporal MC feature to train a novel generalized Ex-NEGAT model. This MC meta-module may be coordinated at certain times (when the cofluctuating links are in a large strength transient) and thus form an *FC*(*t*) module in the conventional sense. This link shows that the form of different meta-modules will fluctuate toward large or weak strengths at different independent times. Importantly, this transient property of the links and their cofluctuations at different time windows seems to be a key feature that plays an important role in the diagnosis task of brain diseases, as demonstrated in this work (Figure 4 and Table 2). Moreover, instead of using the convolution-based method used earlier on FC by Z. Wang et al. (2023), the subgraph creation approach of GNN can aggregate spatiotemporal information and extract node-edge-centric features on the non-Euclidean graph data according to graph topological properties (Scarselli, Gori, Tsoi, Hagenbuchner, & Monfardini, 2009). Our attention-based GNN makes the classification model more transparent and enhances the possibility of detecting potential spatiotemporal imaging biomarkers of ASD. To empirically validate the effectiveness of the attention mechanism in handling noise in rs-fMRI-derived graphs, we conducted a comparative analysis or ablation study by replacing the attention layers in our NEGAT model with a basic GCN that performs uniform aggregation of neighborhood information. We observed a consistent drop in classification performance (8%–10% decrease in accuracy) as reported in Table 6 and Figure 3. These results suggest that the attention mechanism selectively emphasizes more informative edges, thereby enhancing robustness against noisy or less discriminative connections often present in rs-FC data. Our model architecture was designed to be lightweight by minimizing the number of GNN layers and parameters without sacrificing prediction accuracy. This reduction in complexity allows for faster training times and lower memory usage. Furthermore, the MC framework uses simplified graph structures, reducing the need for computationally intensive operations commonly found in alternative approaches.

### Analysis of Each Cluster

In the stratification of ASD subtypes based on MC, our analysis revealed three distinct subgroups within the ASD population. The second subgroup showed a high association with the first and the last subgroup, whereas the first and third subgroups had fewer overlaps with each other regarding the identified meta-links. It is worthwhile to note that while the traditional clustering approach, like the k-means clustering algorithm, directly divides data into clusters, RFCM shows an association between clusters based on shared feature similarity. Based on this analysis, the first and third subgroups contained unique samples, and the samples that came under the first subgroup were more severe to ASD severity. In contrast, samples under the third subgroup were less strongly associated with ASD severity. To get insight into the results, we calculated average MC matrices for both TD and ASD groups (refer to Figure 8). The TD group exhibited hyperconnectivity between edges, while the ASD group showed hypoconnectivity. For each ASD subgroup, we also calculated an average MC matrix. For the third subgroup, connectivity was like the TD group except for a few edges within the DMN and ToM networks, indicating a more significant association with ASD symptom severity. On the other hand, the subgroup with fewer samples showed moderate connectivity, particularly between the DMN, CEN, and ToM networks. The first subgroup displayed hypoconnectivity, especially between the DMN and CEN. Additionally, edges associated with the SN were strongly correlated with other networks across all three clusters. This finding aligns with previous reports of hypercorrelation in the SN among autistics (H. Chen et al., 2017; Margolis, Pagliaccio, Thomas, Banker, & Marsh, 2019).

We also identified an association between clinically identified ASD subtypes (Sadiq et al., 2022) and subgroups identified by the RFCM approach. The clinically defined subgroups were identified mostly based on behavioral scores. Our findings revealed that a majority of individuals with ASD were grouped into Subgroups 1 and 2, which showed stronger associations with ASD symptom severity. Conversely, most individuals with PDD fell into Subgroup 3, which exhibited closer associations with TD groups, indicating milder ASD symptoms. Figure 7 illustrates distinct sample distributions with minimal overlap between the first and third subgroups. Our findings suggest that while clinically defined subgroups share some overlap in MC within specific brain networks, differences in behavioral scores between ATD and APD do not necessarily correspond to differences in functional brain development. Each group showed overlapping patterns in MC. To the best of our knowledge, very few existing frameworks in the literature show an association between clinically identified subgroups and stratified subgroups identified using higher order correlations like MC, which may open a new avenue for studying neurodevelopmental disorders and imaging-derived phenotypes.

### Predicting Symptom Severity in ASD Using MC Matrices and Identifying Relevant ROIs

**Figure 10.**
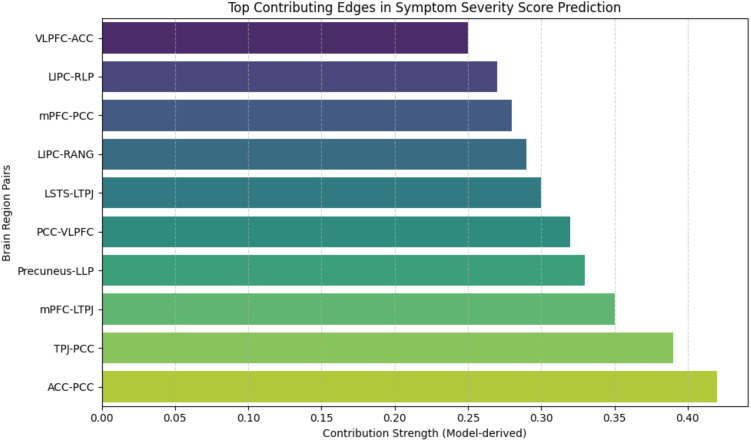
The figure displays the top edges between pairs of brain regions that contribute the most to the prediction of symptom severity score in ASD.

In the previous works (Moradi, Khundrakpam, Lewis, Evans, & Tohka, 2017; Plitt, Barnes, Wallace, Kenworthy, & Martin, 2015), the authors tried to predict symptom severity using FC between ROIs, which relies on spatial features of fMRI data, but there is no proposed framework based on attention-based GNN that predicts symptom severity and diagnostic scores using MC spatiotemporal features. We trained a CPM model based on MC matrices derived from ABIDE I ASD samples and performed testing on ABIDE II samples. Our model significantly predicted ADOS-Total, ADOS-Social, ADOS-Module, ADOS-Communication, ADOS-Stereo, and FIQ scores. We also identified ROIs whose MC edges contributed positively and negatively to the symptom severity score prediction. We found that edges between precuneus, medial prefrontal cortex (MPFC), left superior temporal sulcus, right lateral prefrontal cortex, right superior temporal sulcus, right posterior parietal cortex were contributed as negatively in prediction, whereas all other edges between other ROIs contributed positively (refer to Figure 9). We observed that MC edges consistently influenced prediction outcomes. The connections within and between the ToM, DMN, and SN subnetworks showed the strongest positive contribution, particularly involving the MPFC, posterior cingulate cortex (PCC), and temporoparietal junction (refer to Figure 10). Extant literature suggests that a critical social and cognitive dysfunction in ASD is the impaired ability to decode the mental states, such as beliefs, emotions, and intentions of self as well as others, and altered DMN may be an important neurobiological feature of these deficits (Lombardo et al., 2010). Previous neuroimaging work has consistently suggested that the DMN is one of the most aberrant functional networks in ASD (Glerean et al., 2016; Moseley et al., 2015). Studies of intrinsic FC in ASD reported disrupted within-network connectivity between core DMN nodes (Arbabyazd et al., 2020; Harlalka et al., 2019). To validate the result, we also tried to predict the symptom severity scores at the individual level using DMN, ToM region-specific functional connectivity, but this resulted in fairly lower accuracy on unseen data (Refer to Tables 1 and 2).

We observed that the SN contributed the most to the classification and prediction of ASD. This is consistent with existing literature suggesting the social-brain circuit dominantly includes classic limbic areas, ventral and medial aspects of the prefrontal cortex, the anterior temporal lobes, the PCC, the PCUN, the posterior temporal regions, the temporoparietal junction, the left inferior frontal gyrus involved in social communication, and the somatosensory and anterior insular cortex (Adolphs, 2009; Kennedy & Courchesne, 2008; Roy & Uddin, 2021; Supekar et al., 2022; Wang et al., 2022), which index atypical neurodevelopmental differences in ASD. The proposed framework also reduces computational time and is a lightweight approach in contrast with the whole-brain approach by focusing on a selective set of brain networks and node-edge-centric features; consequently, minimal computational resources are necessary to run the proposed framework. Beyond that, our innovative approach offers explainable AI-based generalized tools to explore the reliable and interpretable neurobiological foundations of various neurodevelopmental and psychiatric conditions, ultimately providing insights into their clinical symptoms and advancing precision neuroimaging in brain disorders.

## MATERIALS AND METHODS

### fMRI Dataset and Preprocessing

In this study, we have used fMRI anatomical and resting-state functional neuroimaging data from the ABIDE I and ABIDE II datasets (Di Martino et al., 2014). The local ethics committee has approved both the initial data collection and the retrospective sharing of a fully deidentified version of the datasets. The functional scan parameters listed per site were utilized for further preprocessing. ABIDE I contains preprocessed images that were used for the study. The data acquired from ABIDE II followed the same processing pipeline as the ABIDE I dataset. The data was preprocessed using the DPARSF (Data Processing Assistant for Resting-State fMRI) toolbox, which is compatible with MATLAB. The first four time points were removed to account for the time taken by the subject to settle down. The reference slice for slice timing correction was set as the slice acquired in the middle of the slice order. The Friston-24 model was employed for head motion correction, and time frames with head motion above 0.2 mm, along with one previous and two following time frames, were added as regressors. Nuisance regression was done using Statistical Parametric Mapping (SPM) a priori masks, and global signal regression was not employed as it is a contentious preprocessing measure. A bandpass filter of 0.01 to 0.1 Hz was applied, and the data was normalized using DARTEL. Smoothing was completed with a Full Width Half Max (FWHM) kernel size of 6 mm. Scrubbing was done for all time frames with a head motion above 0.5 mm and one previous and two following timeframes (after preprocessing: total no. of subjects = 1500, comprised of 711 ASD and 789 TD individuals) (refer to Tables 7 and 8). The configuration for computational processing used in this study was as follows: Graphics Processing Units (GPUs): 16X NVIDIA Tesla V100, NVIDIA CUDA Cores: 81920, NVIDIA Tensor Cores: 10240.

**Table 7.** Demographic information of ASD and TD groups from the ABIDE dataset

**Sr. no.**	**Age (in years)**	**Samples**	**IQ**	**ADI-R Social**	**ADI-R Verbal**
ASD group	*M*: 13.9 ± 6.6, *SD*: 6.6 (range: 6 to 42)	711	105 ± 17	18.8 ± 5.5	15.4 ± 4.5
TD group	*M*: 14.95 ± 5.9, *SD*: 9 (range: 6 to 40)	789	112 ± 14	–	–

**Table 8.** Brain region with MNI coordinates that are associated with theory of mind, default mode network, central executive, and salience networks

**Networks**	**Region of interest**	**MNI coordinates (X, Y, Z)**
SN	Anterior cingulate cortex (ACC)	0, 22, 35
	Left anterior insula (LAINS)	−44, 13, 1
	Right anterior insula (RAINS)	47, 14, 0
DMN	Medial prefrontal cortex (MPFC)	0, 48, −18
	Left lateral parietal (LLP)	−39, −77, 33
	Right lateral parietal (RLP)	47, −67, 29
	Precuneus cortex	0, −49, 40
CEN	Left lateral prefrontal cortex (LLPFC)	−43, 33, 28
	Right lateral prefrontal cortex (RLPFC)	41, 38, 30
	Left posterior parietal cortex (LPPC)	−46, −58, 49
	Right posterior parietal cortex (RPPC)	52, −52, 45
ToM	Posterior cingulate cortex (PCC)	0, −52, 22
	Left superior temporal sulcus (LSTS)	−58, −48, 7
	Right superior temporal sulcus (RSTS)	58, −44, 8
	Left temporoparietal junction (LTPJ)	−58, −58, 22
	Right temporoparietal junction (RTPJ)	58, −52, 20
	Left inferior frontal gyrus (LIFG)	−48, 14, 26
	Right inferior frontal gyrus (RIFG)	48, 14, 26
	Left angular gyrus (LANG)	−46, −72, 31
	Right angular gyrus (RANG)	48, −69, 31
	Left cerebellum (LCEREB)	−20, −72, −36
	Right cerebellum (RCEREB)	20, −72, −36
	Left inferior parietal cortex (LIPC)	−45, −46, 53
	Right inferior parietal cortex (RIPC)	52, −42, 50
	Ventrolateral prefrontal cortex (VLPFC)	42, 46, 0

The ABIDE dataset included (fMRI) data from several sites, leading to heterogeneity caused by engineering and biological sampling biases. We used the Combat harmonization approach to resolve this problem, assuming that the error caused by the imaging features may be corrected by adjusting the position (means) and scale (variances) across the batches. Harmonization was conducted using multivariate linear mixed-effects regression using the following formula (Maikusa et al., 2021):

$$y\left(i,j,v\right)=\alpha \left(v\right)+X^{T}\left(i,j\right)\beta \left(v\right)+\gamma \left(i,v\right)+\delta \left(i,v\right)\epsilon \left(i,j,v\right)$$ (1)

where *α* = average functional volume, *β*(*v*) = *p* * 1 vector of coefficients associated with phenotypic parameters, that is, Age, Gender, ScannerID, X(I, j) = design matrix, p = no. of biological covariates, *ϵ*(*i*, *j*, *v*) = error calculation, *γ*(*i*, *v*) and *δ*(*i*, *v*) = additive and multiplicative of site effect. An empirical Bayesian framework is utilized in ComBat harmonization to compute γ*(*i*, *v*) and δ*(*i*, *v*). The final values that ComBat harmonized are represented as (Maikusa et al., 2021):

$$y_{\left(i,j,v\right)}^{\text{combat}}=\frac{y_{\left(i,j,v\right)}-{\hat{\alpha }}_{v}-X_{\left(i,j\right)}{\hat{\beta }}_{v}-\gamma_{\left(i,v\right)}^{*}}{\delta_{\left(i,v\right)}^{*}}+{\hat{\alpha }}_{v}+X_{\left(i,j\right)}{\hat{\beta }}_{v}.$$ (2)

This method used empirical Bayes to improve the model estimation (Ingalhalikar et al., 2021; Saponaro et al., 2022; Xie et al., 2022; Yamashita et al., 2019). It removed unwanted variation associated with the site and maintained biological association in the dataset.

### Proposed Method for Calculation of MC

#### Calculation of functional connectivity and dFC stream.

We extracted time-series signals from ToM, DMN, CEN, and SN brain networks to calculate MC (refer to Table 8). Based on previous literature, we considered 25 ROIs (Kana et al., 2015). Using Montreal Neurological Institute (MNI) coordinates, we created a spherical binary mask with a 10-mm radius for all selected ROIs and extracted time-series signals. We computed functional connectivity for each individual using Equation 1 (Arbabyazd et al., 2020):

$${\bar{\text{FC}}}_{ij}=\text{Corr}\left[{TS}_{i}\left(t\right),{TS}_{j}\left(t\right)\right],\;\text{with}\left(0\leq t\leq T\right),$$ (3)

where *TS*_*i*_(*t*) and *TS*_*j*_(*t*) are time-series signals from *i*th ROI and *j*th ROI at time *t*, FC_*ij*_ indicates average functional connectivity between ROI *i* and ROI *j*, and *t* represents specific time point within the total time-period *T*. The functional connectivity with pair-wise Pearson correlation matrices was useful for establishing group-level differences.

To track change in functional connectivity patterns over time, we calculated dFC using a sliding window-based approach that computed average FC matrices at different time points. According to the previous study (Arbabyazd et al., 2020), we denoted it as dFC stream. We calculated the temporal frame of dFC stream for each individual (Arbabyazd et al., 2020):

$${FC}_{ij}\left(t_{k}\right)=\text{Corr}\left[{TS}_{i}\left(t\right),{TS}_{j}\left(t\right)\right],\;\text{with}\left(t_{k}\leq t\leq t_{k}+W\right),$$ (4)

where *t*_*k*_ indicates the *k*th temporal frame’s start time and *w* represents the fixed window length. We choose *w* = 20–30 s, according to the literature (Harlalka et al., 2019). Sliding step Δ*τ* is used to separate frame start time, such that *t*_*k*_ = *t*_*k*−1_ + Δ*τ* = *k*Δ*τ*.

#### MC analysis.

Each dFC stream was considered as a collection of time series that defines the time dependency of individual FC pairwise couplings. An *M* * *M* matrix of correlations between the time-dependent strengths of *M* = *N*(*N* − 1) FC links (*N*^2^ pairs of regions, minus the self-loops) was produced by improving the calculation of FC matrix from regional nodes to interregional links (Arbabyazd et al., 2020). The resultant MC matrix expresses the interlink covariance analogous to how the FC matrix explains the internode covariance (refer to Figure 2).

To calculate MC matrices, we used dFC stream and extracted *n* = *N*(*N* − 1)/2 time-series of pairwise FC coupling, in which input was given as *FC*_*ij*_(*t*) for all pair of regions *i* and *j* with *i* < *j* ≤ *N* using Equation 3 (Arbabyazd et al., 2020):

$${MC}_{ij,kl}=\text{Corr}\left[{FC}_{ij}\left(t\right),{FC}_{kl}\left(t\right)\right].$$ (5)

These *MC*_*ij*,*kl*_ entries are computed into a matrix format, making it possible to identify the pair of links contributed to each meta-link directly. Consequently, MC matrices had a dimension of *M* * *M*, where various rows correspond to different directed pairings of regions—that is, both the pair (*i*, *j*) and the pair (*j*, *i*) were included—and only linkages relating to self-loops—that is, of the type, (*i*, *i*) were eliminated from consideration (Arbabyazd et al., 2020). The resulting representation of MC matrices was large since:

$${MC}_{ij,kl}={MC}_{ji,kl}={MC}_{ij,lk}=\ldots ={MC}_{kl,ij}.$$ (6)

MC matrices captured higher level correlation between triplets or quadruplets of ROIs, as compared with FC matrices.

### Node-Edge Connectivity Based Graph Attention Network for Classification

MC matrices are a higher order correlation feature set that tells about edge connectivity between nodes. We have proposed a generalized Ex-NEGAT model that uses an attention matrix by applying a subgraph creation approach, which helped to reduce the execution time of information between the nodes, as well as capture the representation of MC matrices. We provided MC matrices as input to the proposed model. We created a custom plugin that can parse the structure files of these MC matrices. The plugin extracted important characteristics like the computational graph topology and certain node-edge features by partitioning matrices into multiple subgraphs. This subgraph splitting approach saved computing complexity while providing a rich context for the model. The graph representation showed that nodes’ actions might dramatically affect their near neighboring nodes because of their intrinsic interconnection. However, including all neighbors for each node, particularly those at greater distances, was computationally expensive and generally not required. We propose a methodology leveraging a DFS approach to optimize the subgraph depth parameter *d*. The reason behind using the DFS approach instead of the manual selection of the value of *d* was its ability to explore the graph by traversing down one branch as far as possible before backtracking, as well as allowing for dynamic adaptation to the local structure of the graph.

#### Optimization of subgraph depth using dFS.

To optimize the subgraph depth parameter *d*, we employ a DFS algorithm to systematically explore the graph *G*(*V*, *E*) and select subgraphs *G*′(*V*′, *E*′) for each node *n* based on a maximum depth of *d*. The subgraph *G*′ is constructed to include node *n* and its neighboring nodes up to a maximum *d* depth. Here, we selected the value of maximum depth *d* = 5. Formally, let *G*(*V*, *E*) represent the input graph, where *V* is the set of vertices and *E* is the set of edges. For each vertex *n* ∈ *V*, we define *G*′(*V*′, *E*′) as the subgraph containing *n* and its neighbors within a depth limit of *d*. The vertex set *V*′ of *G*′ consists of all nodes within this specified distance, while the edge set *E*′ comprises all edges connecting these nodes. The process begins by iterating over each vertex *n* ∈ *V* in the graph *G*(*V*, *E*). The DFS algorithm explores neighboring nodes up to a maximum *d* depth at each iteration. If the distance from node *n* to a neighboring node *v* is less than or equal to *d*, *v* is added to the vertex set *V*′ of the subgraph *G*′. Similarly, all edges connecting *n* and its neighbors within the specified distance are added to the edge set *E*′ of *G*′. By systematically traversing the graph using DFS and constructing subgraphs *G*′(*V*′, *E*′), for each node *n*, we create a series of subgraphs *G*_*set*_ that capture the local structure and connectivity patterns around each node within the specified depth limit *d*. Within each subgraph, the attention matrix is applied to assign weights to the edges based on their relevance, which is learned during the training phase. This allows the Ex-NEGAT model to focus on the most significant connections, enhancing the interpretability and performance of the network. The attention mechanism ensures that the most informative relationships are given priority, thus reducing noise from less relevant nodes and edges.

#### Model-architecture.

The traditional GNNs were considered only for node features. In the current work, we proposed an attention mechanism for each edge that could consider its multidimensionality and complexity. MC matrices were higher order correlation feature sets that gave correlation values for each edge. In the proposed model, edge features represent the strength of functional relationships between nodes (e.g., connectivity strengths), and these features are treated as distinct from node features, which describe individual properties of ROIs. The model aggregates information from both node and edge features to create a more comprehensive representation of the graph, without merging them into a single entity. The model aggregates edge and node features using a message-passing framework, where edge features influence the update of node representations. Specifically, edge features are incorporated through an attention mechanism, where the importance of each edge is determined based on its strength and contribution to the neighboring nodes. This allows edge features to enrich the node representations while maintaining the distinction between node and edge attributes. We hypothesized that the correlation reflected by edge weight might not represent the attention required for connected nodes. To test our hypothesis, we applied a linear transformation to both node feature set *h*_*i*_ (represents localized activity in brain regions) and edge feature *e*_*ij*_ (captures the functional relationships between regions) set that extracted high-level associated feature set by generating a new feature representation:

$$h_{i}^{\prime }=W_{\text{node}}h_{i}$$ (7)

$$e_{ij}^{\prime }=W_{\text{edge}}e_{ij}$$ (8)

where *W*_*node*_ and *W*_*edge*_ were indicating weight matrices. However, transformed node and edge features were used to calculate separate attention coefficients, where the attention coefficient for node *i* was computed using Equation 7:

$$\alpha_{ij}^{N}=\frac{\text{exp}\left(\text{LeakyReLU}\left({\left(a_{src}^{N}\right)}^{T}h_{i}^{\prime }+{\left(a_{dst}^{N}\right)}^{T}h_{j}^{\prime }\right)\right)}{\sum_{k\in N\left(i\right)}\text{exp}\left(\text{LeakyReLU}\left({\left(a_{src}^{N}\right)}^{T}h_{i}^{\prime }+{\left(a_{dst}^{N}\right)}^{T}h_{k}^{\prime }\right)\right)},$$ (9)

where $a_{src}^{N}$ and $a_{dst}^{N}$ were representing learnable parameters for source and destination nodes and *N*(*i*) = neighbors of node *i*. Similarly, calculated attention coefficient for edge *e*_*ij*_ using Equation 10:

$$\alpha_{ij}^{N}=\frac{\text{exp}\left(\text{LeakyReLU}\left({\left(a_{src}^{E}\right)}^{T}e_{ij}^{\prime }+{\left(a_{dst}^{E}\right)}^{T}e_{ij}^{\prime }\right)\right)}{\sum_{k\in N\left(i\right)}\text{exp}\left(\text{LeakyReLU}\left({\left(a_{src}^{E}\right)}^{T}e_{ik}^{\prime }+{\left(a_{dst}^{E}\right)}^{T}e_{ik}^{\prime }\right)\right)},$$ (10)

where $a_{src}^{E}$ and $a_{dst}^{E}$ were representing learnable parameters for edge features. Finally, we updated the node and edge feature weights according to attention coefficients:

$$h_{i}\prime \prime =\sum_{j\in N\left(i\right)}\alpha_{ij}^{N}h_{j}^{\prime }$$ (11)

$$e_{ij}^{\prime \prime }=\alpha_{ij}^{E}e_{ij}^{\prime }.$$ (12)

Lastly, we concatenated node and edge features and applied a linear layer to reduce dimensionality:

$$h_{i}^{\text{final}}=W_{\text{final}}\left[h_{i}^{\prime \prime }\|e_{ij}^{\prime \prime }\right],$$ (13)

where *W*_*final*_ = weight matrix and $\left[h_{i}^{\prime \prime }\|e_{ij}^{\prime \prime }\right]$ = updated node and edge features.

Finally, we used MLP to make predictions for individual graphs of ASD and TD based on the final feature map $h_{i}^{\text{final}}$. The MLP layers involved two linear layers with dropout = 0.2 and a ReLU function following the first linear layer to improve the robustness of the proposed model.


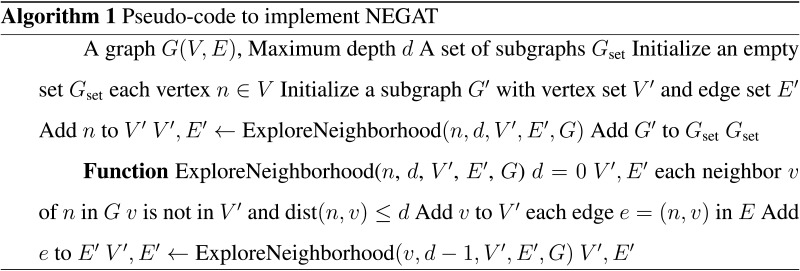


#### Training and testing process.

We trained our model in different ways, in which the learning rate was set to 0.0001, the batch size was 64, and the number of epochs was set to 100. The L2 regularization parameter has been set to 0.0001 for all the linear layers to prevent overfitting. We utilized the Adam optimizer and cross-entropy loss as a loss function. In addition, to prevent the issue of the model overfitting, we use a technique called label smoothing, as illustrated in the Equation 12 (Müller, Kornblith, & Hinton, 2019):

$$y_{k}^{LS}=y_{k}\left(1-\alpha \right)+\frac{\alpha }{K},$$ (14)

where *K* = 2, that was indicating number of class, *y*_*k*_ ∈ (0, 1) = true labels, $y_{k}^{LS}$ = corresponding smoothing label, and *α* = 0.1. The loss function was calculated using Equation 13:

$$\text{Loss}\left(y,p\right)=\beta \|W{\|}_{2}+\sum_{k=0}^{K}-y_{k}^{LS}\text{log}\left(p_{k}\right),$$ (15)

where *W* = weight of linear layer, *y* = real label (after label smoothing), *p* = predicted label, *p*_*k*_ = predicted probability of class *k*.

#### Classification evaluation and experimental settings.

The performance of the proposed model was tested in three different ways to make it more robust, explainable, and generalizable: (a) we trained the model on ABIDE I dataset and performed testing on ABIDE II dataset; (b) we performed fivefold cross-validation across all the subjects; and (c) we also used leave-one-out approach in which, we trained model on complete ABIDE I + II except one site, and perform testing on that site data as an independent dataset. We repeated this process for all sites, including ABIDE I and II datasets. We also trained different types of GNN models like GCN, Chebyshev Convolution, Residual GCN, and GraphSAGE to cross-check the proposed model’s performance using MC matrices. The approach was applied uniformly across all models during training to ensure consistency in model comparisons. Label smoothing, which was applied to all models with the same smoothing factor, helped to regularize the models by reducing overfitting in predictions and improving generalization. To validate the novelty of the proposed MC feature set, we also performed classification using FC and dFC matrices.

### Identification of ASD Subgroups Using RFCM Algorithm

We subjected MC matrices of each individual to an unsupervised RFCM algorithm to identify subgroups presented within the ASD population (Mitra, Banka, & Pedrycz, 2006; Raja, Sasirekha, & Thangavel, 2020; Shi, Lei, Zhou, & Gong, 2016). The reason behind using RFCM was the ability of fuzzy set to handle overlapping partitions by applying gradual membership degrees to each sample in each cluster and the ability of Rough set to deal with uncertainty and incompleteness, *in which a centroid and two approximated regions were represented as two clusters*. (Shi et al., 2016; Zhou, Pedrycz, & Miao, 2011). The detailed methodology of the RFCM algorithm has been added to the Supporting Information.

### Symptom-Severity Score Prediction for ASD Samples

We identified the association between neurobiological features, that is, MC matrices and symptom severity scores. We investigated whether resting-state MC could identify ASD symptom severity scores for each individual. For this purpose, we used CPM, in which each participant’s MC matrix and behavioral scores were subjected to the CPM model (Shen et al., 2017). CPM performs regression-based statistical significance tests to identify positive and negative correlations between connectivity patterns and the target outcome. This process generates a set of features (edges) that are positively or negatively correlated with the prediction target. Previously, the authors used the CPM approach to behavioral scores using node-centric features like functional connectivity (Bhavna et al., 2023; Shen et al., 2017). There is no such framework in which the CPM approach is implemented using an edge-centric feature, that is, MC matrices. The initial step involves splitting the input data into training and testing sets. In this study, we trained the model on ABIDE I ASD samples and performed testing on ABIDE II ASD samples. Subsequently, across all participants in the training set, each connection in the connectivity matrices is associated with the behavioral measures using various linear regression techniques, such as Pearson’s correlation, Spearman’s correlation, or robust regression. After the linear regression, the most significant connections were singled out for further analysis. Typically, prominent connections were selected through statistical significance testing, that is, chosen connections with correlation values exceeding a predefined threshold (0.4 with *p* < 0.05). For each participant, the most vital connections are consolidated into a single summary value. This is commonly done by summing the strengths of the connections. Next, a predictive model is constructed, assuming a linear relationship between the summary value of connectivity data (independent variable) and the behavioral variable (dependent variable). Subsequently, summary values are computed for each participant in the testing set, and these values are input into the predictive model.

## CONCLUSION

In the current study, we proposed a lightweight, explainable, and generalized attention-based GNN framework that successfully distinguished individuals with ASD from TD individuals using MC as a feature set within the multisite ABIDE I and ADIDE II datasets by leveraging ToM, DMN, CEN, and SN brain networks with remarkable accuracy. We also identified subgroups within ASD samples using the RFCM approach and predicted each individual’s social symptom severity scores, thereby paving the way for potential future applications in individual profiling. There is no such framework in the literature that could give such a considerable performance on large unseen datasets using an edge-centric feature set. Our proposed framework is novel in the following ways: (a) use of MC as an edge-centric feature set for classification and symptom-severity score prediction; (b) consideration of only four brain networks with 25 ROIs that reduce computational power and make it lightweight; (c) unique architecture of a generalized Ex-NEGAT model; (d) performance on a large unseen testing dataset compared with the training dataset; and (e) identification of subgroups within ASD samples and prediction of symptom-severity scores. There are also certain limitations, such as (a) we tested our framework only on resting-state MC; there is a need to test it during a cognitive task, and (b) a longitudinal dataset needs to be considered. In the future, we will work on it. In summary, our discovery of robust, individualized functional brain connectivity biomarkers within the relevantly mentioned brain regions in ASD can revolutionize our understanding of the causes, diagnosis, and targeted modulation of connectivity in pervasive neurodevelopmental disorders. Beyond that, our innovative approach offers Ex-AI-based generalized tools to explore the reliable and interpretable neurobiological foundations of various neurodevelopmental and psychiatric conditions, ultimately providing insights into their clinical symptoms and advancing precision neuroimaging in brain disorders.

## ACKNOWLEDGMENTS

We acknowledge the generous support of IIT Jodhpur Core funds and the Computing facility. DR acknowledges the generous support of the NBRC Flagship program BT/MEDIII/NBRC/Flagship/Program/2019: Comparative mapping of common mental disorders (CMD) over the lifespan.

## SUPPORTING INFORMATION

Supporting information for this article is available at https://doi.org/10.1162/NETN.a.32.

## AUTHOR CONTRIBUTIONS

Km Bhavna: Conceptualization; Data curation; Formal analysis; Investigation; Methodology; Software; Validation; Visualization; Writing – original draft; Writing – review & editing. Niniva Ghosh: Data curation; Formal analysis; Investigation; Methodology. Romi Banerjee: Investigation; Methodology; Project administration; Resources; Software; Supervision; Writing – original draft; Writing – review & editing. Dipanjan Roy: Conceptualization; Data curation; Formal analysis; Funding acquisition; Investigation; Methodology; Project administration; Resources; Software; Supervision; Validation; Visualization; Writing – original draft; Writing – review & editing.

## FUNDING INFORMATION

The author(s) declare that no financial support was received for the research, authorship, and/or publication of this article.

## DATA AVAILABILITY STATEMENT

The raw datasets used in this study is publicly available from ABIDE and preprocessed data generated during the analysis pipelines in the present study are available from the corresponding author upon reasonable request. The codes for all the analyses carried out in this paper are also available at GitHub: https://github.com/dynamicdip/.

## Supplementary Material



## TECHNICAL TERMS

Autism: A neurodevelopmental disorder characterized by deficits in social cognition and self-related impairments, speech and communication deficits, repetitive and restricted behaviors, and sensory motor abnormalities.
Dynamic functional connectivity: Time resolved functional connectivity estimated from fMRI BOLD signals to capture large-scale brain network dynamics.
Meta-connectivity: Meta-connectivity quantifies how fluctuations in different functional connectivity (FC) links covary over time. Conceptually, it captures the higher order temporal coordination between dynamic FC patterns, offering a richer spatiotemporal representation of brain network organization beyond traditional static or even first-order dynamic FC.
Graph attention neural network: These are subclasses of graph neural networks (GNNs) designed to operate on graph-structured data by leveraging attention mechanisms assigning different weights to different nodes and edges based on their relevance to target nodes rather than treating them equally.
Rough fuzzy c-means algorithm: Rough fuzzy c-means (RFCM) is an advanced clustering algorithm that integrates fuzzy rough set theory with traditional Fuzzy C-Means (FCM) to enhance cluster recognition in uncertain or ambiguous data. This can be applied to discover autism subtypes.
Typical development: Development of large-scale functional brain networks, for example, theory of mind (ToM), default mode network (DMN), central executive network (CEN), and salience network (SN) in neuroimaging studies associated with social cognition, communication, and cognitive flexibility.
