# Supplementary material for: A lightweight, end-to-end explainable, and generalized attention-based graph neural network model trained on high-order spatiotemporal organization of dynamic functional connectivity to classify autistics from typically developing
Source: Netw Neurosci. 2025 Nov 20;9(4):1323–51. doi: 10.1162/NETN.a.32 (PMC12635838; doi:10.1162/NETN.a.32)
Supplement: Supplementary file 1 [file netn-9-4-1323-s001.pdf]

Supplementary Material: A Lightweight, End-to-End Explainable, and Generalized attention-based Graph Neural Network model trained on high-order spatiotemporal organization of dynamic functional connectivity to classify Autistics from Neurotypicals

Km Bhavna[1], Niniva Ghosh[2], Romi Banerjee[3], and Dipanjan Roy[4]

[1,3]Department of Computer Science and Engineering, Indian Institute of Technology, Jodhpur, Rajasthan, India

[2,4]School of Artificial Intelligence and Data Science, Centre for Brain Science and Application, Indian Institute of Technology, Jodhpur, Rajasthan, India

[4]Cognitive Brain Dynamics Lab, National Brain Research Centre, Manesar, India

May 22, 2025

1 Methodology

1.1 Identification of ASD Sub-groups using Rough Fuzzy C-Means Algorithm

1.1.1 Background

A fuzzy set A in population X is a class of events defined by continuous membership degree and membership function $\mu_A(x)$ (Shi et al., 2016). The value of $\mu_A(x)$ is associated with an interval [0,1] that indicates the degree of membership of x in A. The membership function can be defined using the following equation:

$$\mu_A(x) : X \rightarrow [0, 1] \tag{1}$$

Where $\mu_A(x)$ represents the weighted coefficient that reflects vagueness in decision-making on the belonging of x (Shi et al., 2016). Assume $x \in X$, and $\mu_A(x) = 1$ is indication higher degree for x belonging to A, whereas $\mu_A(x) = 0$ representing lower degree.

A rough set is used to reduce vagueness in a given set by applying some approximation. Let's assume U = nonempty set, R = equivalent relation on U, then U can be partitioned into disjoint classes of R:

$$\frac{U}{R} = \{X_1, X_2, ..., X_m\} \tag{2}$$

Where X represents the equivalence class of R, $i = 1, 2, ..., m$. Using the obtained uncertain information, it is tough to describe X in real applications. One way to resolve it is to use lower and upper approximations in a given arbitrary set $X \in 2^U$ using the following equation:

$$\underline{R}(X) = \bigcup_{X_i \subseteq X} X_i; \quad \bar{R}(X) = \bigcup_{X_i \cap X \neq \emptyset} X_i \tag{3}$$

Where the upper approximation $\bar{R}(X)$ is defined as an object having a nonempty intersection with X, whereas the lower approximation $\underline{R}(X)$ represents objects that are a subset of X.

1.1.2 RFCM Architecture

To identify no. of sub-groups within ASD samples, we implemented RFCM in which cluster was represented using fuzzy lower approximation and fuzzy upper approximation, and centroid was updated using the following way:

$$v_i = \begin{cases} w * A_1 + w'^{*B_1} & \text{if } \underline{R}_1(\beta_i) \neq \emptyset,\ R_{1b}(\beta_i) \neq \emptyset \\ A_1 & \text{if } \underline{R}_1(\beta_i) \neq \emptyset,\ R_{1b}(\beta_i) \neq \emptyset \\ B_1 & \text{if } \underline{R}_1(\beta_i) \neq \emptyset,\ R_{1b}(\beta_i) \neq \emptyset \end{cases} \quad (4)$$

$A_1 = \dfrac{1}{n_i} \sum_{X_j \in \underline{R}_1(\beta_i)} (\mu_{ij})^m X_j;\ n_i = \sum_{X_j \in \underline{R}_1(\beta_i)} (\mu_{ij})^m$
$B_1 = \dfrac{1}{n_j} \sum R_{1b}(\beta_i)(\mu_{ij})^m X_j;\ n_j = \sum_{X_{j1b}(\beta_i)} (\mu_{ij})^m$

Here, c represents the number of clusters, β_i represents cluster i for $i = 2, \ldots, c$, $\underline{R}_1(\beta_i)$ is the lower approximation, $R_{1b}(\beta_i)$ is the boundary region, μ_{ij} is the fuzzy membership value, X_j is the data point, w is the weight for the lower approximation, w^* is the weight for the boundary region, and m is the fuzzy coefficient. If neither of two approximations was empty, then both the lower approximation and the boundary region were used to compute a new centroid. The relative significance of two approximations is determined by weighted parameters (w and w'). Since the lower approximation contributes more to the computation of a new centroid than the boundary region. $0 < w' < w < 1$ and $w + w' = 1$. Fuzzy membership degrees were used to identify which approximations an item belonged to. With the addition of fuzzy sets, the rough set-based clustering method could handle overlapping situations more effectively. The fuzzy membership function has the advantage of simultaneously making an item belong to all clusters with varying degrees of ownership, improving the capability in dealing with uncertainty caused by overlapping boundaries.

Algorithm 1 Rough Fuzzy C-Means (RFCM) Clustering Algorithm

Require:
- Meta-connectivity data X (size $M \times M$)
- Number of clusters c
- Fuzzy coefficient m ($m > 1$)
- Weighted parameters w and w' ($0 < w' < w < 1$ and $w + w' = 1$)
- Maximum iterations max_iter
- Convergence tolerance tol

Ensure: Cluster assignments for each subject, cluster centroids

```
Initialize fuzzy membership matrix μ randomly such that Σ_{i=1}^{c} μ_ij = 1 for all j
Initialize iteration counter to 0
Initialize convergence flag to false
while iteration counter < max_iter and convergence flag is false do
    Increment iteration counter
    for each cluster i do
        Compute lower approximation A_1:
```

$$A_1 = \frac{1}{n_i} \sum_{X_j \in \underline{R}_1(\beta_i)} (\mu_{ij})^m X_j$$

```
        Compute boundary region B_1:
```

$$B_1 = \frac{1}{n_j} \sum_{X_j \in R_{1b}(\beta_i)} (\mu_{ij})^m X_j$$

```
        Update centroid v_i:
```

$$v_i = \begin{cases} w \cdot A_1 + w' \cdot B_1 & \text{if } \underline{R}_1(\beta_i) \neq \emptyset \text{ and } R_{1b}(\beta_i) \neq \emptyset \\ A_1 & \text{if } \underline{R}_1(\beta_i) \neq \emptyset \text{ and } R_{1b}(\beta_i) = \emptyset \\ B_1 & \text{if } \underline{R}_1(\beta_i) = \emptyset \text{ and } R_{1b}(\beta_i) \neq \emptyset \end{cases}$$

```
    end for
    Store old membership matrix μ_old = μ
    for each data point X_j do
        for each cluster i do
            Update membership value μ_ij:
```

$$\mu_{ij} = \left(\sum_{k=1}^{c} \left(\frac{d(X_j, v_i)}{d(X_j, v_k)} \right)^{\frac{2}{m-1}} \right)^{-1}$$

```
        end for
    end for
    Check for convergence:
    if ||μ − μ_old|| < tol then
        Set convergence flag to true
    end if
end while
Assign each data point X_j to the cluster with the highest membership value μ_ij
return Cluster assignments, cluster centroids =0
```

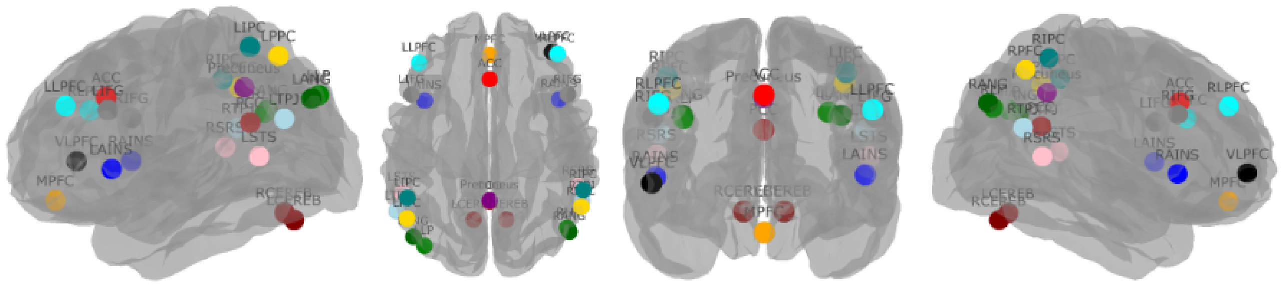

Figure S1: The figure illustrates the selected ROIs.

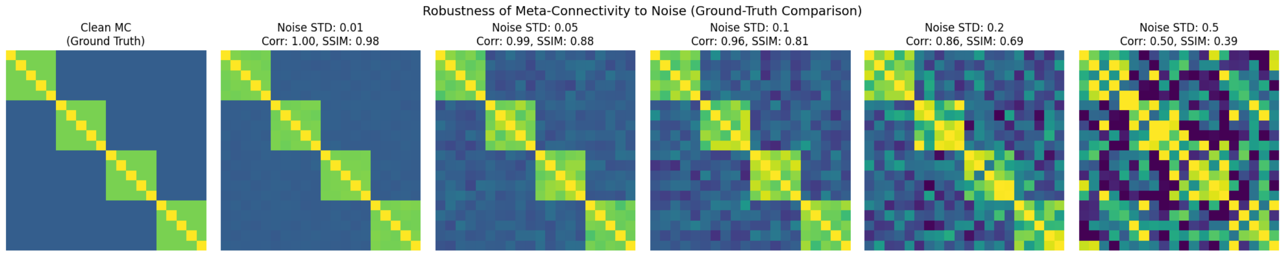

Figure S2: Robustness analysis of the Meta-Connectivity (MC) framework under increasing noise levels. The leftmost panel shows the clean, structured ground-truth MC matrix with clustered connectivity patterns. Subsequent matrices show the effect of adding Gaussian noise with increasing standard deviation (STD). Pearson correlation and Structural Similarity Index (SSIM) values are reported to quantify similarity with the ground truth. Results demonstrate that MC retains significant structure under low to moderate noise (STD 0.2), supporting its robustness in noisy data scenarios.

2 Results

2.1 Computation of Meta-Connectivity Matrices

To assess the robustness of the Meta-Connectivity (MC) matrices to noise, Gaussian noise with varying signal-to-noise ratios (SNRs), ranging from 20 dB to -5 dB, was added to the data. MC matrices were then computed from sliding-window dynamic functional connectivity (dFC) matrices. The similarity between the noise-free and noisy MC matrices was evaluated using Pearson correlation and the Structural Similarity Index Measure (SSIM). A controlled simulation was conducted in which structured ground-truth MC matrices were systematically perturbed with increasing levels of Gaussian noise. Results showed that the MC matrices retained their core structure and clustering features under low to moderate noise levels (standard deviation = 0.01–0.2), with Pearson correlation values 0.86 and SSIM values 0.69. However, significant degradation of the MC matrices was observed at higher noise levels (standard deviation = 0.5).

2.2 Prediction of Symptom Severity Score

To identify the robustness of the model in predicting symptom severity scores, we analyzed both the distribution of prediction errors and summary error metrics. The violin plots demonstrate that the majority of prediction errors are tightly centered around zero with minimal skewness, indicating that the model is neither systematically over- nor under-predicting any score. This reflects low bias and stable performance across samples. Complementing this, the MAE and RMSE values remain consistently low across all severity scores, confirming that the model achieves high predictive accuracy with limited variance and minimal influence from outliers. Together, these results demonstrate that our model generalizes well and performs reliably in estimating symptom severity.

References

Jiao Shi, Yu Lei, Ying Zhou, and Maoguo Gong. Enhanced rough–fuzzy c-means algorithm with strict rough sets properties. *Applied Soft Computing*, 46:827–850, 2016.

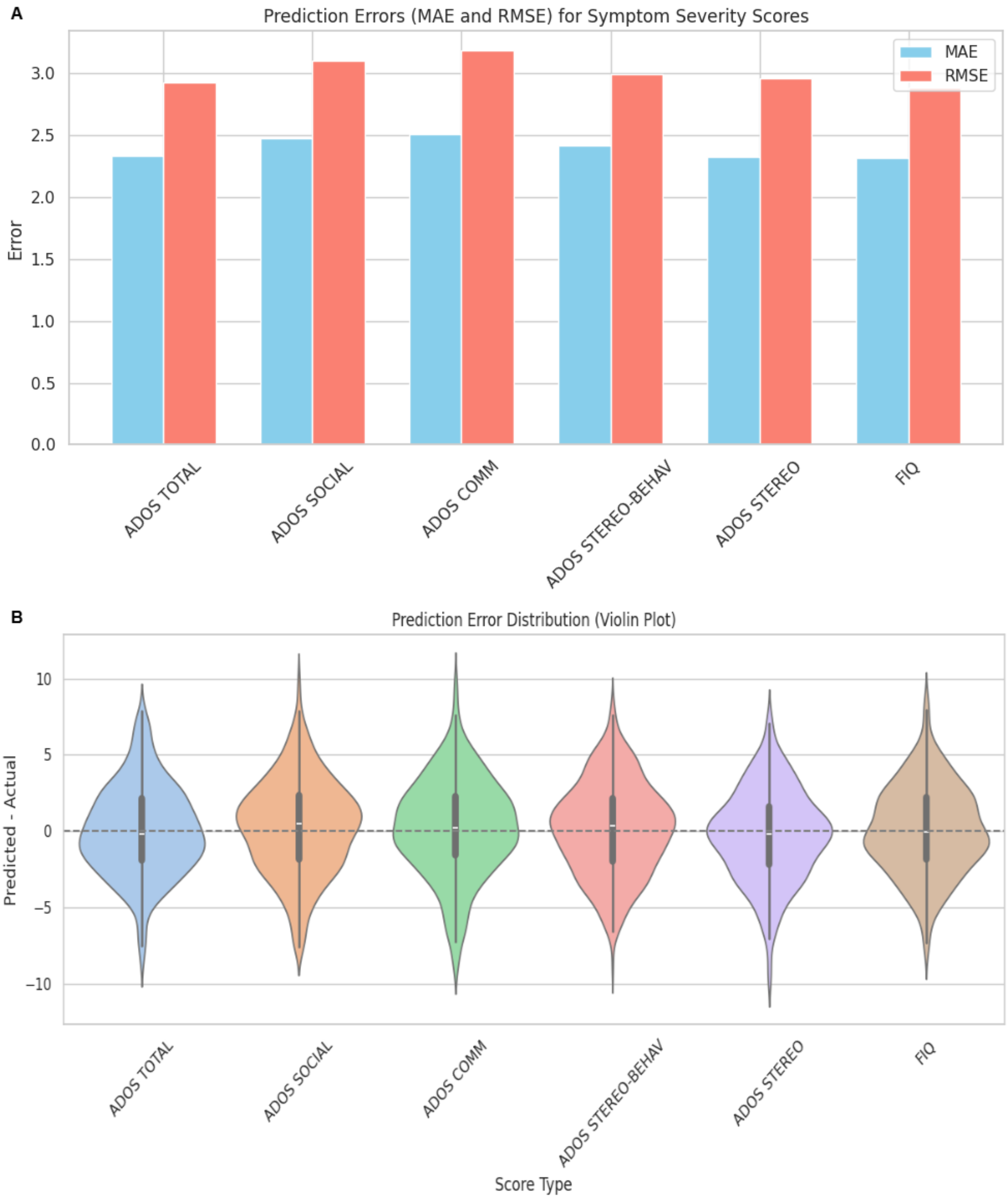

Figure S3: Prediction performance for symptom severity scores. **A)** Bar plots depict the mean absolute error (MAE) and root mean squared error (RMSE) for each score, summarizing pre-diction accuracy and sensitivity to outliers. **B)** Violin plots show the distribution of prediction errors (Predicted - Actual) across different scores, illustrating bias and variance.
